# Critically appraised topic on adverse food reactions of companion animals (1): duration of elimination diets

**DOI:** 10.1186/s12917-015-0541-3

**Published:** 2015-08-28

**Authors:** Thierry Olivry, Ralf S. Mueller, Pascal Prélaud

**Affiliations:** Department of Clinical Sciences, College of Veterinary Medicine, North Carolina State University, 1060 William Moore Drive, Raleigh, NC 27607 USA; Medizinische Kleintierklinik, Centre for Clinical Veterinary Medicine, Ludwig Maximilian University, Veterinärstrasse 13, 80539 Munich, Germany; Clinique Advetia, 5 rue Dubrunfaut, 75012 Paris, France

**Keywords:** Allergy, Atopic dermatitis, Canine, Cat, Dog, Elimination diet, Feline, Food allergy, Itch, Pruritus

## Abstract

**Background:**

Restrictive (i.e. elimination)-provocation dietary trials remain the standard of care to diagnose cutaneous adverse food reactions (CAFRs) in dogs and cats. There is currently no consensus on the duration of elimination diet trials that would permit the highest sensitivity of diagnosis of CAFR in companion animals.

**Results:**

The search for, and review and analysis of the best evidence available as of December 14, 2014 suggests that, by 5 weeks in dogs and 6 weeks in cats after starting an elimination diet, more than 80 % of patients had achieved a remission of clinical signs of CAFR. Increasing the diet trial duration to 8 weeks leads to a complete remission in more than 90 % of dogs and cats with CAFR.

**Conclusions:**

For diagnosing CAFRs in more than 90 % of dogs and cats, elimination diet trials should last at least 8 weeks.

## Background

The current standard for the diagnosis of cutaneous adverse food reactions (CAFRs) in dogs and cats involves the performance of dietary restriction-provocation tests. Over time, the recommendations for optimal duration of restrictive dietary trials (i.e. “elimination diets”) have varied from 3 to 12 weeks; there is currently no consensus on the duration of such trials for optimal diagnosis of CAFRs.

### Clinical scenario

Your patient is a 2-year-old male castrated German shepherd dog that is very itchy. On examination, you notice erythematous macules, patches and papules on the abdomen, axillae and the perineum; it also suffers from intermittent diarrhea. As this dog is already on an optimized flea control regimen, you suspect that food reactions cause all these signs. You would like to perform an elimination dietary trial to confirm this. You wonder how long this elimination diet should last.

### Structured question

In dogs and cats suspected of having a CAFR, how long should an elimination diet trial last for highest sensitivity of diagnosis?

### Search strategy

The CAB Abstracts and Web of Science (Science Citation Index Expanded) databases were searched on December 16, 2014, using the following string: ((dog or dogs or canine) or (cat or cats or feline)) and (food or diet*) and (reaction or allerg* or hypersensitivity) and (trial or restriction or elimination) and (skin or cutaneous or itch or pruritus). We limited the search to 25 years (1980 to 2014) and we excluded congress proceedings notes and book chapters.

### Identified evidence

Our literature search identified 108 and 78 citations in the CAB Abstract and Web of Science databases, respectively; 45 articles were common to both databases. Abstracts were read and pertinent articles were read in full. Two types of original articles provided data relevant to the question of interest: these articles either reported large case series of dogs or cats with CAFRs or the effect of feeding one or more test diets to companion animals with CAFRs. Among these papers, five [1–5] and three [1, 3, 6] respectively provided specific data on the time needed for manifestations of CAFR to improve in dogs and cats fed a restrictive diet.

### Evaluation of evidence

In the selected articles, the diagnosis of CAFR was generally made in dogs and cats with nonseasonal pruritus after the exclusion of other relevant causes of pruritus and a complete or marked (at least 50 %) reduction of clinical signs after feeding a restrictive (elimination) diet, the latter consisting of novel or partially hydrolyzed ingredients. Cases with partial reduction of itch were then confirmed as having CAFR after recurrence of signs after provocation with previous diets or ingredients.

**Fig. 1:**
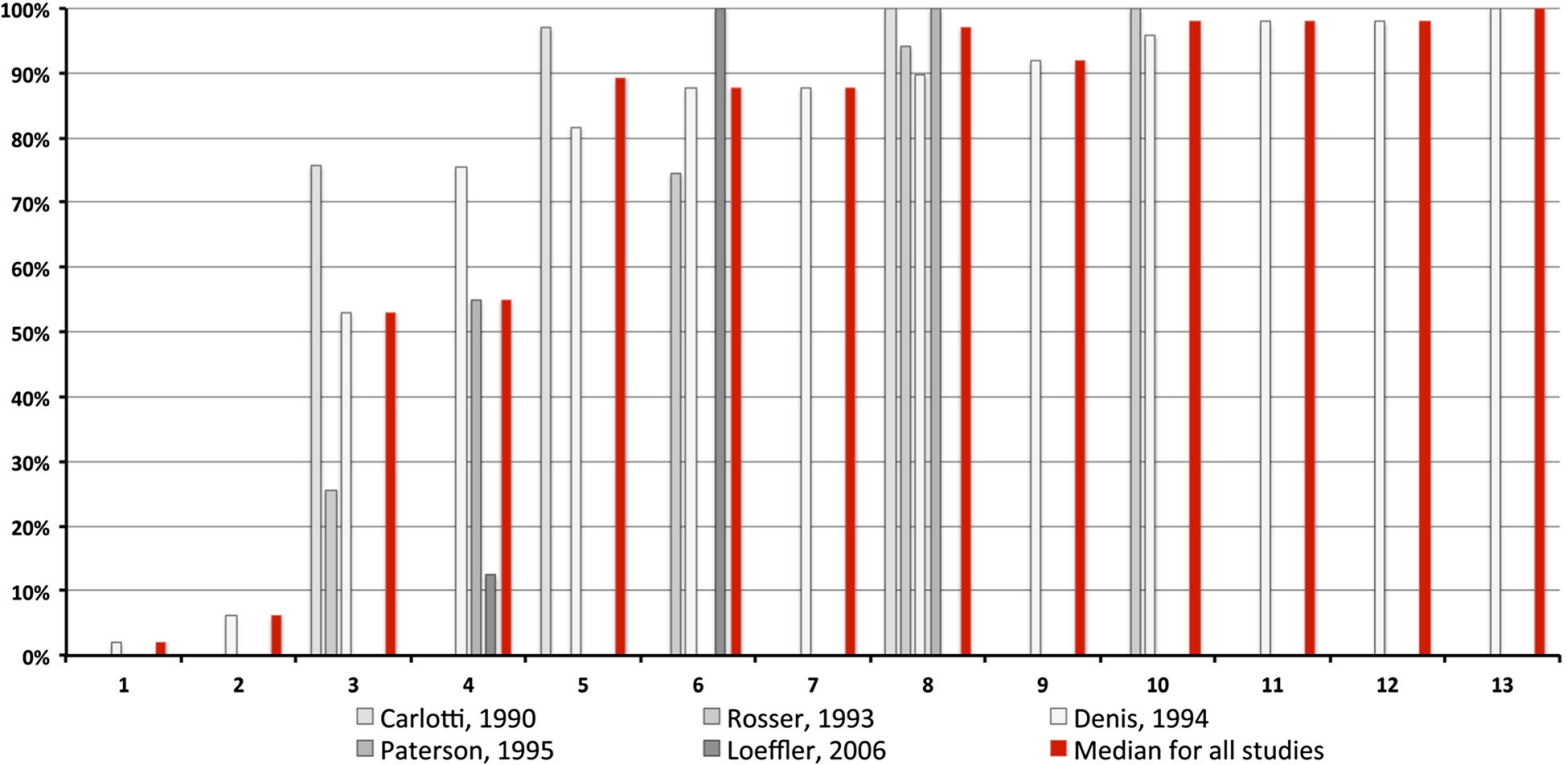
Cumulative percentages of clinical remission in 209 dogs with CAFRs over time (in weeks)

The cumulative percentages of complete, or near complete, remission of clinical signs of CAFR in dogs fed a restrictive diet are depicted on Fig. 1. Based on the information gathered from 209 dogs with CAFR, we can estimate that, after 3 weeks of a diet change, approximately half of dogs had achieved a marked reduction of their signs. From 5 weeks onward, signs had returned to normal in more than 85 % of dogs, and this percentage increased to over 95 % if extending the dietary trial to 8 weeks. Fewer than 5 % of dogs needed an elimination diet of up to 13 weeks for a complete remission of signs of CAFR to occur.

**Fig. 2:**
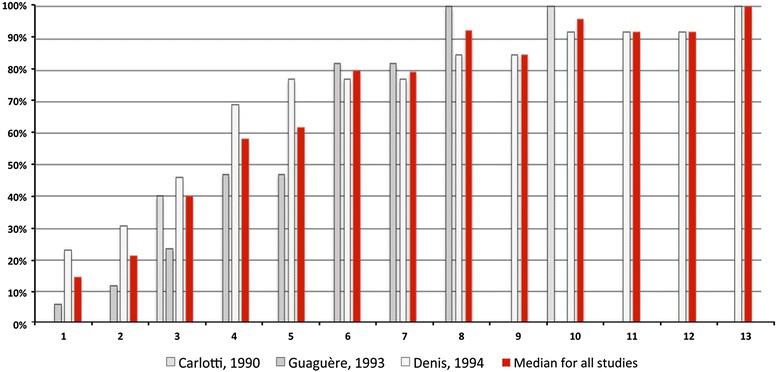
Cumulative percentages of clinical remission in 40 cats with CAFRs over time (in weeks)

Cumulative percentages of clinical sign remission in 40 cats with CAFR are illustrated on Fig. 2. It took approximately 4, 6 and 8 weeks of a restriction diet for 50, 80 and 90 % of cats to achieve a remission of their clinical signs, respectively.

### Conclusion and implication for practitioners

To diagnose CAFR in at least 80 % of dogs and cats, a restrictive (elimination) dietary trial should last a minimum of 5 weeks in dogs and 6 weeks in cats. Increasing the duration of the restrictive diet to 8 weeks will increase the sensitivity of diagnosis to more than 90 % of cases in both species.

The ultimate goal of an elimination diet is to enable the positive confirmation of a CAFR with a provocation with suspected food items. As a result, veterinarians might elect to perform provocation tests soon after a remission of signs is achieved in a patient, even if such remission were to happen before 8 weeks after starting the elimination diet.

## Abbreviations

CAFR: Cutaneous adverse food reaction
CAT: Critically-appraised topic
